# Chronic mechanical irritation and oral squamous cell carcinoma: A systematic review and meta-analysis

**DOI:** 10.17305/bjbms.2021.5577

**Published:** 2021-12

**Authors:** Archana A. Gupta, Supriya Kheur, Saranya Varadarajan, Sameena Parveen, Harisha Dewan, Yaser Ali Alhazmi, Thirumal A. Raj, Luca Testarelli, Shankargouda Patil

**Affiliations:** 1 Department of Oral Pathology and Microbiology, Dr. D. Y. Patil Dental College and Hospital, Dr. D. Y. Patil Vidyapeeth, Pune, India; 2 Department of Oral Pathology and Microbiology, Sri Venkateswara Dental College and Hospital, Chennai, India; 3 Department of Maxillofacial Surgery and Diagnostic Sciences, College of Dentistry, Jazan University, Jazan, Saudi Arabia; 4 Department of Prosthetic Dental Sciences, College of Dentistry, Jazan University, Jazan, Saudi Arabia; 5 Department of Maxillofacial Surgery and Diagnostic Sciences, Division of Oral Pathology, College of Dentistry, Jazan University, Jazan, Saudi Arabia; 6 Department of Oral and Maxillofacial Sciences, Sapienza University, University of Rome, Rome, Italy

**Keywords:** Chronic trauma, carcinogenesis, oral squamous cell carcinoma, risk factor

## Abstract

The objective of the present article was to qualitatively and quantitatively review the association between chronic mechanical irritation and oral squamous cell carcinoma (OSCC). PubMed, SCOPUS, and Web of Science databases were searched using the keyword combinations “chronic trauma and oral squamous cell carcinoma; chronic irritation and oral squamous cell carcinoma; chronic irritation and oral cancer; and chronic trauma and oral cancer.” Duplicates and irrelevant articles were excluded after the title and abstract screening. The full texts of the remaining articles were assessed using selection criteria. A total of 375 (PubMed-126; SCOPUS-152; WOS-97) articles were screened, and 343 duplicates and irrelevant articles were excluded from the study. Only 9 of the remaining 32 articles met the selection criteria and were included in the qualitative analysis. Buccal mucosa and tongue, being highly prone to chronic irritation through the dental prosthesis, were the common sites for OSCC. Edentulous subjects with ill-fitting dentures were at a high risk of developing chronic irritation associated-OSCC. According to the Joanna Briggs Institute of risk assessment, eight of the nine included studies had a low risk of bias. The quantitative analysis showed a significant association (p < 0.00001) between the chronic oral mucosal irritation and OSCC with an overall risk ratio of 2.56 at a confidence interval of 1.96-3.35. Chronic oral mucosa irritation has a significant association with OSCC, and the nature of association could be that of a potential co-factor (dependent risk factor) rather than an independent risk factor.

## INTRODUCTION

Oral squamous cell carcinoma (OSCC) the sixth most common neoplasm of the head and neck regions accounts for 80-90% of malignancies of the oral cavity [1]. Around 300,000 new cases of oral cancer have been reported worldwide with 145,000 deaths [2]. The incidence of the disease is variable worldwide with a higher incidence rate in developing countries. The highest incidence and mortality rates have been reported in Southeast Asia [3]. In India, it accounts for 30% of all malignancies contributing to a mean age distribution of 55 years in the adult population [4]. OSCC generally arises in middle-aged and older people with a slight male predilection although the incidence in the female population has increased in recent years. Clinically almost all oral cancers, except those in the earliest stages occur as ulcers with an indurated margin. The tongue is the most common site of occurrence of OSCC. Despite several recent advances in therapeutic strategies, the 5-year survival rate has not seen much improvement in recent years [5].

The multifactorial etiology of OSCC includes tobacco, alcohol, and betel quid with and without added tobacco as the major risk factors [6]. In addition to these known risk factors, several associated risk factors have been suggested for OSCC. These include microbes, diet, socioeconomic status, and occupational carcinogenic substances, etc. Factors having a controversial role with limited and inconsistent evidence in OSCC etiology include ethnicity and race, oral hygiene and dentition, environmental, genetic, marijuana smoking, khat chewing, nicotine replacement therapy, HIV infections, and alcohol in mouthwashes [7-10]. Among these, much importance is given to microbes, while the other potential risk factors remain relatively unexplored.

One such poorly-explored factor is chronic mechanical irritation (CMI). There have been cases reported with OSCC developing in an oral site with a history of CMI. The CMI may result from poor oral hygiene, [11] poor dentition, [12] missing teeth, [13], and prosthetic factors. Prosthetic factors associated with CMI are intra-oral prosthesis which is ill-fitting, have sharp/rough surfaces, or lack retention stability. Dental factors associated with CMI include mal-positioned, sharp/fractures/rough surfaces of natural teeth. Functional factors associated with CMI include parafunctional habits such as cheek biting and tongue thrusting [14-18]. Mucosal pathologies due to CMI are related to its intensity and duration which range from frictional keratosis in mild conditions to fibrous hyperplasias in moderate to severe conditions [19-21]. Considering the high morbidity and mortality rates of OSCC, strategies to prevent the disease is the need of the hour. Carcinomas associated with tobacco-related habits can be prevented by counseling and de-addiction programs, however, the other risk factors such as microbes, chronic trauma can be prevented by routine dental examination and adequate prophylactic measures.

Before exploring into adapting prophylactic measures, it is vital to confirm the association between CMI and OSCC. The lack of conclusive evidence for the same can be largely attributed to the presence of confounding factors in most of the studies. The inclusion of inflammation as the seventh hallmark of cancer since 2009 [22] had led to the exploration of factors capable of inducing inflammation-mediated carcinogenesis. Concerning the oral mucosa, the dental, and the prosthetic factors capable of inducing CMI were assessed closely for their potential role in OSCC. A strong association between ill-fitting dentures and OSCC was found in a systematic review published in 2017 [23] and a meta-analysis published in 2014 (odds ratio [OR]: 3.90, 95% confidence interval [CI]: 2.48-6.13) [24]. Although there are two previous meta-analyses about the relationship between CMI and OSCC, since the last of these was carried out in 2017, the present review was conducted to assess the current update on the association of CMI with OSCC. A narrative review published in 2018 [25] assessing CMI as a potential causative factor in OSCC using the Bradford Hill criteria of causation summarized that there was a significant limitation in all the 22 studies included in their review for any conclusive inference. Thus, despite several original studies and qualitative and quantitative assessment, the association between OSCC and CMI remains largely inconclusive. The present article was formulated to answer two queries. First, is there a significant association between the CMI and OSCC? Second, if there is an association is that association of a causal nature?

## MATERIALS AND METHODS

### Search strategy

The International prospective register of systematic reviews (PROSPERO) was searched for systematic reviews assessing the potential associating between OSCC and CMI, following which the present review was submitted for registration. Preferred Reporting Items for Systematic Reviews and Meta-Analysis Protocols (PRISMA) were strictly adhered to Shamseer et al. [26].

The review was conducted in 3 steps:

· Keyword combinations “Chronic Irritation and Oral Squamous Cell Carcinoma, Chronic Irritation and Oral Cancer, Chronic trauma and Oral Squamous Cell Carcinoma and Chronic trauma and Oral Cancer” were used in SCOPUS, PubMed, and Web of Science databases to identify potential articles
· The titles and the abstract of the identified articles were screened by AAG and SK for relevance and potential duplicates
· The full text of the article selected through screening was assessed by AAG and SK using the following selection criteria:

### Inclusion criteria

Original research studies published in the English language assessing the potential association between CMI and OSCC.

### Exclusion criteria

Reviews, short articles (commentary, letters, correspondence), case reports, experimental animal models, and articles published in languages other than English.

Step 2 and 3 were conducted by two reviewers (AAG, SK). Kappa coefficient (k) was calculated for steps 2 and 3 to determine the inter-observer reliability.

Vital data including the aim of the study, the sample characteristics, nature of trauma, sample size, the results including the odds ratio, and the inference were retrieved from the included studies.

### Risk of bias

Joanna Briggs Institute’s methodology for cross-sectional studies and case control studies was used to assess the risk of bias in the included studies [27,28].

## RESULTS

### Study selection

**FIGURE 1:**
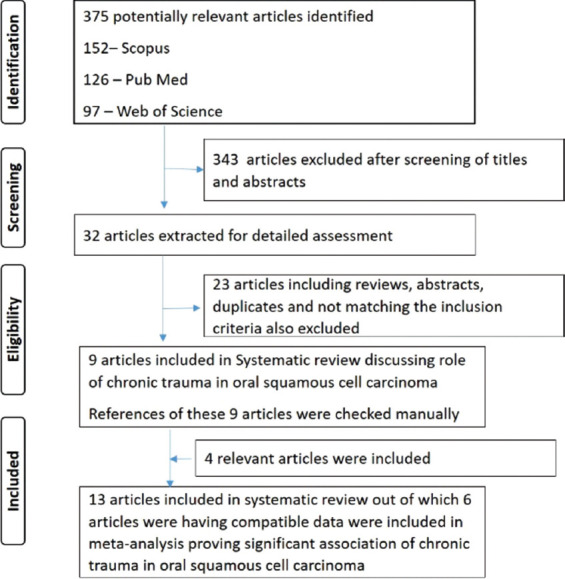
Summary of the search strategy employed in the review.

A total of 375 articles including 126 from PubMed, 152 from Scopus, and 97 from Web of Science were retrieved using the keywords. Screening the titles and abstracts of the identified articles revealed that 343 articles were either duplicate or were not related to the topic of interest and thus were excluded from the study. The full text of the remaining 32 articles was assessed using the selection criteria. Only 9 fulfilled the eligibility criteria and were included in the systematic review. Cross references of these 9 articles were checked manually and another four articles were included which gave a total count of 13 articles that were included in the systematic review. The workflow of systematic review has been summarized in Figure 1. k value of 0.98 and 0.96 was obtained for the 2^nd^ and 3^rd^ step of the review process indicating a good overall inter-observer reliability. Table 1 summarizes the data extracted from the included studies.

**TABLE 1:**
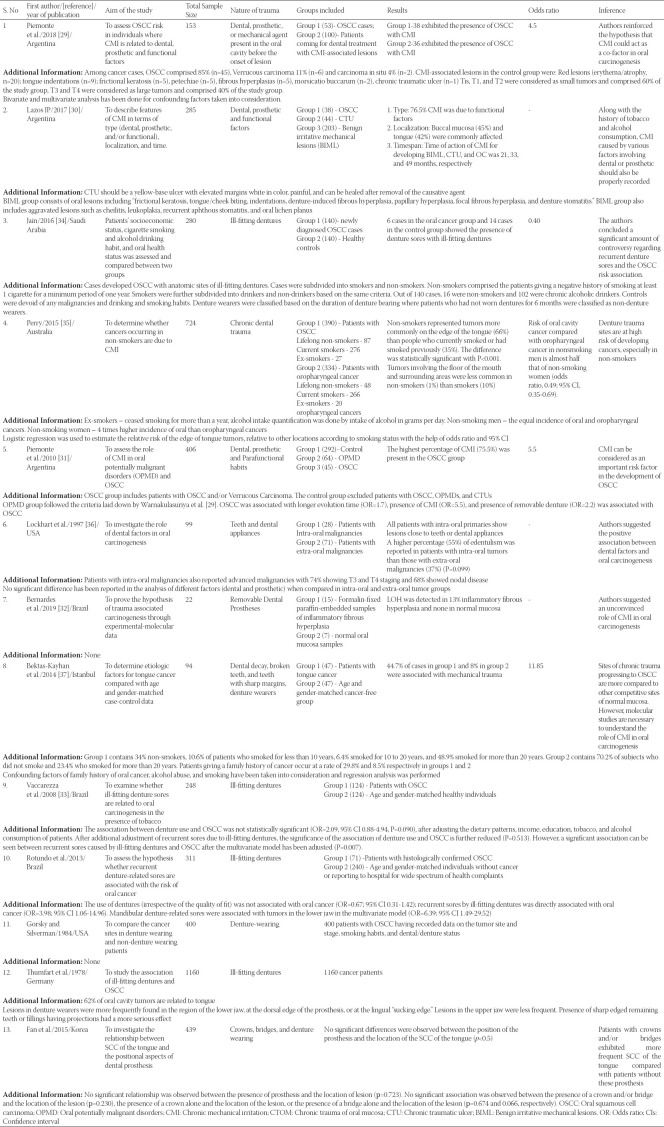
Data extracted from the studies included in the qualitative review

### Studies characteristics

Of the studies included in the review, 3 were from Argentina [25,29,30] and Brazil [31-33], 2 were from USA [34,35], and one each from Australia, Saudi Arabia, Turkey, Germany and Korea [36-40]. All the included studies assessed the role of chronic trauma as a risk factor in OSCC. The articles included in the present review were case-control cross-sectional studies. The comparison group in the studies included normal oral mucosa; chronic traumatic ulcer; benign irritative mucosal lesions; and extra-oral OSCC.

### Qualitative analysis of the role of chronic trauma in oral carcinogenesis

Despite using different research models and designs, all the included studies reported a significant association between CMI and OSCC. Most lesions occurred on the buccal mucosa and tongue which are the most common sites to be injured by dental, functional, and prosthetic factors. A higher percentage of edentulism and ill-fitting dentures have been associated with OSCC [34]. In non-smokers, OSCC presented most commonly on the lateral margin of the tongue which was the most frequent site for chronic trauma [35]. The presence of CMI-associated lesions in OSCC cases further reinforced the association between CMI and OSCC [29].

### Quantitative analysis

**FIGURE 2:**
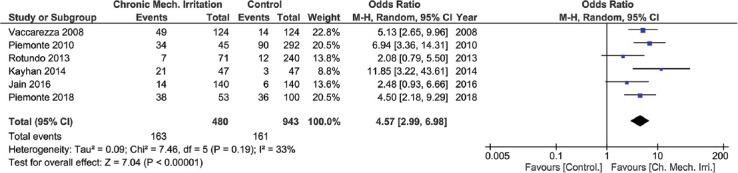
Forest plot summarizing the quantitative analysis.

Of all the included studies, only 6 had data compatible with a meta-analysis. The individual risk ratio and CI of each of the 6 studies were estimated, based on which an overall odds ratio of 4.57 at a CI of 2.99-6.98 was determined (Figure 2). The quantitative analysis based on the odds ratios from the 6 studies showed that CMI holds a significant association (*p* < 0.00001) with OSCC (Figure 2).

### Risk of bias

**FIGURE 3:**
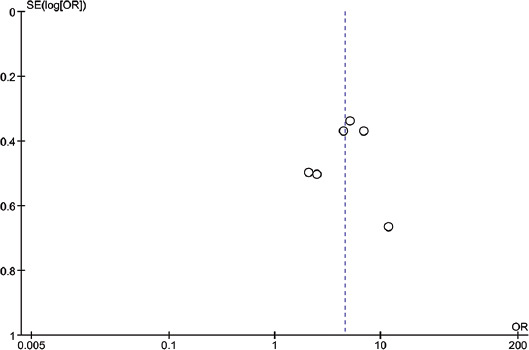
Funnel plot summarizing the publication bias among the selected articles.

Studies were assessed separately for bias based on the 8 criteria of the Joanna Briggs Institute for cross-sectional studies and 10 criteria of the Joanna Briggs Institute for case-control studies. Five of the six studies included in the case-control studies have taken confounding factors into consideration [25,30,32,33,38]. The rest of the parameters including the reliability of the method (to measure exposure and to study the outcomes) and application of statistical analysis were accounted for in most of the studies. Among the 7 studies included in the qualitative analysis, 5 were shown to have a low risk of bias and 2 with high risk of bias according to Joanna Briggs Institute methodology. Similarly, all the 6 studies included in the meta-analysis had a low risk of bias. Tables 2 and 3 summarize the risk of bias assessment of the included studies. Publication bias among the selected references has been shown in the funnel plot as Figure 3. There is a symmetrical scatter of points on both sides of weighted average mean difference which indicates no publication bias. Furthermore, points are not just clustered at the bottom but they are scattered throughout the funnel. Hence, standard error is small or sample size in the studies is not small.

**TABLE 2:**
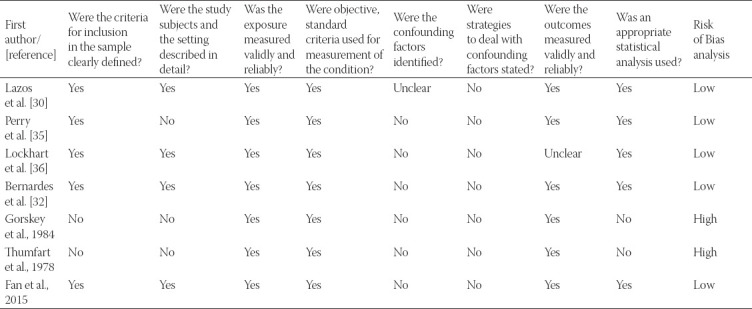
Risk of bias analysis (Joanna Briggs Institute methodology) of the studies included in the qualitative analysis

**TABLE 3:**
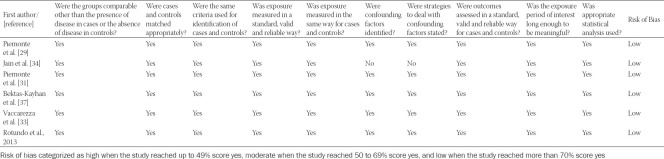
Risk of bias analysis (Joanna Briggs Institute methodology) of the studies included in the Quantitative analysis

## DISCUSSION

The role of inflammation in carcinogenesis is often underplayed, despite its inclusion as the 7^th^ hallmark of cancer since 2009 [23]. CMI of the oral mucosa is the result of repeated, low-intense action of an oral deleterious agent such as sharp teeth, ill- fitting dentures, and functional alterations, separately or in combination causing sustained trauma [41]. There are three types of CMI factors: Dental (malpositions, sharp/broken teeth, and/or rough or defective restorations); prosthetic (ill-fitting dentures, rough/sharp/overextended flanges, and lack of retention/stability); and functional (swallowing, occlusal, and other dysfunctional disorders) [30]. Thus, it is plausible that the chronic inflammation caused by intra-oral factors such as ill-fitting dentures, sharp teeth could be associated with increased risk of oral cancer. Thus, the present article was formulated to qualitatively and quantitatively review original studies evaluating the potential association between CMI and OSCC.

CMI with or without associated factors such as tobacco and alcohol was found to exhibit a significant correlation with OSCC [29]. Considering the effect of tobacco on oral mucosal immunity, Johnson et al. in their *in-vitro* study have reported that nicotine increases the secretion of inflammatory cytokines IL-1, IL-6, IL-8, TNF and McP-1 gingival keratinocytes and hGFs [42]. Furthermore, in the epithelial cells, tobacco causes reduction in epithelial barrier function, reduction of mucosa secretion, alteration of cytokine production, alteration of several receptor ligand expression reduces barrier function, increases mucus production, modifies cytokine/chemokine production, alteration of receptor/ligand, reduction in phagocytic activities, increased inflammation, and lymphocyte function [43]. Thus, it can be inferred that tobacco exposure on a chronically irritated mucosa tobacco can aggravate inflammation further thereby promoting carcinogenesis. Since alcohol is synergistic with tobacco in oral carcinogenesis, the same effect on CMI could be expected. Chronic irritation of oral mucosa may interfere with the oral microbiome, thus causing an imbalance in oral homeostasis. Pang et al. have reported the link between the oral microbiome, the epithelial barrier, the immune system, and chronic inflammation in an oncogenic parallelogram [44]. Furthermore, the altered oral microbiome drives the chronic inflammation that may precede OSCC, and alters host cell response. CMI is also a conditioning element of HPV infectious cycle and oncogenesis. It causes increased expression of syndecan 1 by the basal keratinocytes thereby increasing the ability for HPV attachment and internalization which is vital for persistent infection, viral replication, and oncogenesis [45]. Considering the minor risk factors such as diet, consumption of spicy food may aggravate chronic irritation and a diet deficient in antioxidants and anti-inflammatory agents would promote oxidative stress thereby promoting carcinogenesis. Thus, these mechanisms explain the fact that individuals with CMI may have an increased risk of tumors even in other parts of the mouth than that directly affected by the CMI. Furthermore, a time-dependent association between CMI and OSCC was reported with an odds ratio of 1.7 [30]. The mean duration of CMI associated with OSCC was found to be 49 months [29]. Hence, it can be inferred that the increase in duration of untreated CMI is associated with an increased risk for developing OSCC. The dental factors such as sharp teeth, malocclusion, and prosthetic factors such as ill-fitting denture and lack of stability had a strong association with OSCC [29,34]. Lockhart et al. [34] reported that all intra-oral malignancies arose at the areas in contact with teeth and/or appliances. This could be attributed to the fact that inflammatory cells are vital constituents of the tumor microenvironment thereby promoting cellular proliferation, cell survival, invasion, and metastasis [34]. About 74% of intra-oral tumors associated with CMI were found to be T3 or T4 (TNM staging) lesions. This could be attributed to the inflammatory response facilitating carcinogenesis as a result of CMI caused by dental and prosthetic factors. However, these results have to be viewed with caution as tumor size is highly influenced by the delay in diagnosis, which makes it very difficult to establish an association between tumor size and inflammation by CMI with the observational data. CMI could play a role in the progression of OSCC, even if it did not exert an initiating or promoting causal role. Hence, future studies that specifically analyze the size of the tumor in relation to the presence of CMI, adjusting the statistical analysis according to other parameters, fundamentally delay in diagnosis have to be conducted to establish the role of CMI in cancer progression. Studies by Jain et al. [36] and Vaccarezza et al. [32] reported the association of OSCC with ill-fitting dentures. Furthermore, they reported the association between recurrent sores and OSCC.

Considering the site, 60% of the OSCC in non-smokers frequently occurred on the lateral border of the tongue which is the most common site of dental trauma [37]. Thus, it is important to screen the sites prone to CMI for early signs of malignancy [38]. On the contrary, Bernardes et al. [31] reported a lack of significant correlation between chronic trauma and oral cancer, which in turn could be due to the smaller sample size and unequal distribution of cases and controls. Furthermore, the study has several limitations including the fact that the study has analyzed only para-prosthetic fibrous hyperplasias and the same cannot be generalized to all lesions associated with CMI and clarification on whether these hyperplasias were previously treated and the inflammatory state of each case is unclear. The study has not reported an odds ratio with respective CI. Hence, the results of this study have to be interpreted with caution considering the bias in the study and lack of statistical correlation. In addition to the reported case-control studies, the association of CMI with OSCC has been reported in *in-vivo* animal models. Perez et al. [46] demonstrated carcinogenic potential in oral chronic traumatic ulcers (CTU) induced due to constant irritation by dental edges or restorations. In the experiments, conducted in animal models, CTU was noted to be an accelerating factor for exo-endophytic tumors.

Although there is substantial qualitative and quantitative evidence to suggest the association of CMI with OSCC, there is little evidence on whether CMI could be an independent risk factor for OSCC. Most of the published data have reported CMI as a cofactor or a promotor of OSCC. This hypothesis has been reinforced by Piemonte et al. in 2010 and 2018 [25,30]. Studies done in experimental animal model have shown that CMI at least has a promoter effect, however, unlike experimental studies in animals, observational studies in humans cannot control all the real variables that affect patients, and there may be undetected carcinogenic factors in certain individuals, with which CMI can interact.

Even, without being mutagenic, CMI could generate conditions that could facilitate initiation. Gilligan et al. in their cross-sectional study have reported that several specimens of CTU expressed altered immunohistochemical pattern similar to OSCC thereby confirming the role of CMI in oncogenesis [47]. Studies done on the experimental animal model showed that when combined with chronic trauma, a relatively lower dose of 7,12-dimethyl-benzanthracene (DMBA) was sufficient to induce carcinogenesis. Thus, CMI promotes the carcinogenic ability of known carcinogens. Despite promoting DMBA’s carcinogenic potential, chronic trauma independently was not shown to be capable of initiating carcinogenesis. In the study by Perez et al. 2005 [46], 6 experimental groups were established. Although the trauma as the only variable did not generate carcinomas neither did the DMBA applied in initiating doses. Thus, it can be understood that, initiation and promotion separately, do not cause cancer. However, in one of the models of their study, they had applied DMBA as initiator, to which was additional doses of DMBA was given combined with trauma. In this model, they reported that 18 of 18 animals developed carcinomas, a higher percentage than the model with the highest application of DMBA without trauma, and in turn, the carcinomas appeared earlier (lower latency), at the trauma site (possibility of detecting the site of the appearance of carcinoma), and with more aggressive clinical and histological characteristics. Therefore, the combination of trauma with another initiator carcinogen was more harmful than that of the initiator alone [46]. Other studies in animals have also reported similar results.

In addition to oral cancer, the pro-carcinogenic association of inflammation has not also been reported in tumors of other sites. These include the association of melanoma with inflammation of the skin, carcinoma of bladder associated with inflammation of the bladder, pancreatic carcinoma with chronic pancreatitis, hepatocellular carcinoma with hepatitis C viral infection (chronic viral hepatitis), cervical carcinoma with chronic HPV infection, colorectal carcinoma with inflammatory bowel disease, and carcinoma of the lung with inflammation of lung induced by smoking [48]. Chronic inflammation results in the production of multiple factors such as infiltration of leukocytes, production of cytokines such as tumor necrosis factor-alpha (TNF-alpha), interleukins and chemokines such as cyclooxygenase-2 (COX-2), matrix metalloproteinases (MMP), reactive oxygen, and nitrogen species (ROS and RNS), and an activated nuclear factor B (NF-B). These factors promote carcinogenesis by stimulation tumor growth and modulation of the tumor microenvironment. The four major pathways of modulation are by promoting angiogenesis, facilitating the proliferation of malignant cells thereby promoting tumor growth, by suppressing immune surveillance, and by inhibiting apoptosis. Studies have proposed that rather than genetic changes CMI leads to epigenetic changes inhibiting DNA reparation and apoptosis [49]. Thus, CMI could potentially be a promoter for OSCC rather than an initiator [50]. The above evidence reinforces the need for evaluation of intraoral and prosthetic factors that could cause CMI in addition to the other known risk factors such as tobacco and alcohol.

The narrative review by Piemonte and Lazos used the Bradford Hill Criteria to provide an in-depth assessment of the association between CMI and OSCC. Given the conflicting data, the authors concluded by citing the various shortcomings of their included studies and suggested that future studies provide a clear definition for CMI. The authors had also advised recording the factors that could be considered as triggers for CMI including dental, prosthetic, and functional, especially in OPMD, OSCC cases. The need for exploring the biological pathways of CMI associated with OSCC was also emphasized [25]. The present review also faced similar restrictions as most of the included studies despite a low risk of bias, did not account for confounding factors. Considering whether inflammation as a result of CMI could be an independent risk factor in oral carcinogenesis, it has to be understood that in a multifactorial disease setting, a promoter carcinogen could be considered a potentiating rather than a triggering cause. Furthermore, as previously described in multistep carcinogenesis process, initiation alone does not produce OSCC, and requires promotion subsequent to initiation. Therefore, the triggering factor could be the promoter factor, with the initiating factor being a predisposing cause. Thus, CMI can also be considered an effect modifier or enhancer, and hence controlling this factor in high risk population can reduce the incidence, morbidity and mortality rates of OSCC. In other words, CMI could interact with other factors, acting as a component cause of a sufficient cause and by eliminating that component cause, the effects of said sufficient cause are controlled or diminished. Although not all OSCCs are associated with cancer similar to tobacco or alcohol, defining CMI as a dependent factor could result in an undervalued interpretation of the role of CMI in carcinogenesis. The fact that it is a dependent factor does not imply that the implementation of treatment and prevention protocols for CMI could not positively impact on reducing the risk of OSCC.

Hence, future research with clear study design, appropriate sample size, and appropriate adjustment of the statistical analysis according to other parameters has to be conducted to establish the role of CMI in cancer progression.

## CONCLUSION

The present article qualitatively and quantitatively reviewed the association between CMI and OSCC. Studies with different study designs have shown a common consensus in the associating CMI with OSCC. However, due to the presence of confounding factors and the lack of long-term prospective human studies, it was not possible to assess CMI as an independent risk factor for OSCC. Based on the meta-analysis, CMI has a significant association with OSCC, but the association could potentially be of a co-factor (dependent risk factor) rather than an independent risk factor. Further multicenter prospective clinical studies including patients without known confounding (risk) factors would allow the assessment of CMI as a potential independent risk factor for OSCC.
